# Bright’s Disease of the Kidney

**Published:** 1864-02

**Authors:** John Bartlett

**Affiliations:** Chicago


### ARTICLE VII.

### BRIGHT'S DISEASE OF THE KIDNEY.

By JOHN BARTLETT, M.D., of Chicago.

Read before the Chicago Medical Society.

Mrs. C. E., aged thirty-one years, of good constitution and of temperate habits, fell under my observation on the 24th of March last.

The following is her history: Thirteen years since, after the birth of her first child, she had a severe attack of dyspepsia, lasting for three years, and reducing her much in strength and flesh. She suffered also from leucorrhœa. As a remedy for these difficulties, soda, and the muriated tincture of iron were taken habitually. At the expiration of three years, her health was nearly or quite restored.

Five years since, she gave birth to a child. In this labor, her attendant having given assurance that delivery should be accomplished by a certain hour, gave her some medicine "to make his words good." The labor was actively stimulated, and the child violently extruded. Since then, she has been troubled with uterine disease, which was aggravated two years since, by a miscarriage, and again by an abortion in October last. For some time past, there has been a recurrence of her dyspeptic symptoms, the most prominent being acidity of the stomach and meteorism.

The patient was of large frame, and somewhat corpulent. She was four months advanced in pregnancy. On the 10th of March last, while lifting a weight, she felt something tear within her. Flooding and fever followed, on the 22d and 23d, the hæmorrhage was excessive; on the 27th, abortion occurred, the functions of the uterus being perfectly performed.

This fever, thus set-up, apparently by accident, and terminating in death in sixty-three days, it is my object to describe. The Society will bear in mind that my observation of the case dates two weeks after its commencement; and it may here be mentioned that the notes from which this report is made were taken as an aid in the treatment only: they are consequently incomplete.

The symptoms may be considered in three stages. The first occupying twenty-five days; the second thirteen days, and the third twenty-five days. These were severally stages of progression, of mitigation, and of aggravation. The most prominent symptom, throughout, was fever. The febrile condition determined the state of the patient, and there was a general coincidence between it and the other symptoms in mildness or severity. It began with daily flushes of heat; these increasing in severity, became distinct paroxysms of rigor and fever. On the 12th day the fever was constant, though slight. There was a sensation of chilliness in the morning, and an evening exacerbation. In the progress of the disease the rigors were for a time lost sight of: the fever was continued. By the twenty-fifth day, the pulse, always frequent, had become rapid and feeble. The skin was invariably warm and dry, till the coming on of exhaustion at the close of the first stage, when the hands and face were cool. The tongue, at first coated with a white fur, soon became dry and brown; the teeth being covered with sordes. Wakefulness in the beginning, gave place to nervousness, and this to hallucination and delirium; finally there was an approach to stupor. The face had become pale, sallow, and death-like. The bowels were torpid, yielding small, dark-colored and offensive discharges, when stimulated by enemata. There was some tympanites. At the close of this stage of progression there occurred a new difficulty, which, in the end, proved most distressing and dangerous. The patient was seized with a violent attack of dyspnœa. There was an accumulation of a quantity of tenacious mucus in the air passages, producing great embarrassment of respiration, and causing a well-nigh fatal exhaustion. The dangerous depression resulting from this attack being recovered from, mitigation of the symptoms followed, the fever gave evidence of gradual abatement. The pulse lost its great frequency, and increased in force and volume. The skin losing much of its heat, was occasionally moist and natural. The tongue cleared completely; the sleep was satisfactory, and listlessness gave place to sprightliness. The expression was excellent; the bowels became less torpid and their secretions natural. Appetite and strength returned in good degree. For discouragement, there remained accelerated respiration, and occasional attacks of dyspnœa; but these were not frequent, and the respiratory movements daily approached the proper mean.

At the end of seventeen days, improvement ceased. Every evening brought a chill, with its accompanying aggravation of fever. The pulse again became frequent and feeble. The skin, hot and dry during the fever, was at other times unnaturally moist. There was no natural sleep; there was nervousness and occasional headache, delirium, and general subsultus tendinum. The difficulty of breathing was constant, and the paroxysms of dyspnœa frequent. The appetite and strength failed. Death occurred by asthenia.

Having thus described the symptoms generally and collectively, it will be profitable, in order to the full understanding of the significance of each, that a review of the more important should be taken. Here, also, will be noticed certain important signs of disease, not mentioned in the general description. To such, in particular, I invite the attention of the Society.

The first peculiarity to be noticed is the frequency and want of equality of the pulse. In the first twelve days of my observation, twenty records, taken at all hours, give the average number of pulsations in the minute as 142. In the stage of mitigation, the average number was 126; and in the last period the average was 138. The mean of seventy-five observations throughout the disease gives a pulse of 134. The want of equality in the pulse was not less striking than its great frequency. In one instance, the pulsations at 4, A.M. were 113; at 6, A.M., 176; at 8, A.M., 128. Thus a variation of from twenty to sixty beats to the minute, in a few hours, was not uncommon, and the variation as to volume, force, and quickness was almost as remarkable as the irregularity in the number of beats.

An inquiry as to what hour in the twenty-four, the pulse was the more or less frequent, gives this result:

| | | | |
|---|---|---|---|
| For the morning, - | 124 | For the evening, - | 139 |
| For noon, - - | 136 | For midnight, - | 136 |

The relation of the movements of the heart to those of the chest was quite constant throughout. Thus, the extreme ratio of the number of pulsations to the number of respiratory acts, in the minute, was, for the minimum, as twenty-two to ten; for the maximum, as forty-five to ten. This coincidence may be further established thus. The average number of respiratory movements was:

| | | | |
|---|---|---|---|
| For the morning, - | 36 | For the evening, - | 47 |
| For noon, - - | 44 | For midnight, - - | 42 |

It will be observed that the movements of the chest, like those of the heart, were lowest in the morning, gradually increased at noon, and reached a maximum in the evening; falling toward midnight to the same number (nearly) as at midday.

Cerebro-spinal symptoms came on early. Dilitation of the pupils existed throughout the third week of disease; it was not again noticed until two days before death. No connection between this and any other condition could be traced. Next followed hallucinations, hilarious, and busy delirium, listlessness, stupor, and indifference. The dyspnœa was in part a nervous phenomenon. Under this head may be mentioned a general tremor or agitation of the body, occurring in the last week of life. This vibration was especially striking in parts moved in respiration. Some days before death, she had, both waking and sleeping, spasmodic startings of parts or of the whole of the body, as if from an electrical shock.

The bowels were usually costive, though diarrhœa was easily induced. In general, when one discharge occurred, several followed; and they were frequently, whether the quantity was small or large, consistent or otherwise, followed by great exhaustion. The discharges were usually semi-consistent, sometimes entirely so, sometimes watery. There was occasional colic, and meteorism was almost constant in the last half of the disease. No other connection between the state of the bowels and other symptoms was noticed than this; exhaustion after an operation, lead sometimes to a paroxysm of dyspnœa. On the contrary, in the latter stages, the embarrassment of the lungs was relieved by an evacuation of the bowels.

Acceleration of the respiration was noticed as early as the twenty-fifth day of illness. Three days after this, the first paroxysm of difficulty of breathing took place. These attacks of dyspnœa were frequently simply a consequence of an accumulation of mucus in the air tubes; the excitor or motor power being deficient from exhaustion, from sleep, or from stupor. At other times they seemed dependent upon nervous influence. They occurred at all hours, most commonly at night, sometimes with distinct periodicity. During the paroxysms, tenacious, glairy mucus was expectorated. The average number of respiratory movements per minute during these attacks was fifty-two. They varied much in severity, occasionally being so slight that a change of position, or a cough, would bring relief. At other times they lasted for hours, threatening the life of the patient. Toward the termination of the case, the difficulty of breathing was constant. For two days before death, there was what might be called the respiration of the dying, The average number of respiratory acts per minute, from fifty observations taken in the last forty days of life was forty.

Notwithstanding this long-continued difficulty of breathing, no physical sign of disease in the chest was discovered until the sixth week, when dulness on percussion, and fine crepitation in the right infra scapula and dorsal regions indicated engorgement of corresponding portions of the lung.

On the forty-third day œdema of the hands and face first attracted attention. But, upon reflection, it was evident that this symptom had existed for some time unnoticed. For a week or more, extraordinary changes had been marked in the expression. Pallor and thinness of the countenance would offer at one visit a bad augury of the case. The next day dropsy would have filled out the face, and fever enlivened it, so as to give to the patient the appearance of health. These changes were mostly due to the œdematous condition. The anasarca was slight, and not constant. It was confined almost entirely to the face and extremities. It came and went without regularity, and without appreciable change in the general state of the patient.

In a retrospective glance at this case, several symptoms are noted which should have led to an earlier examination of the urine. Inquiry elicited these facts, having reference to the kidneys: Pain of the back was complained of once, on the twenty-fourth day. Nine months previously, the patient had received an injury. In descending the stairs she fell, striking the loins against a step with great violence. The effects of this fall were traced up to the time of this sickness in a sense of weakness in the lumbar region, necessitating the use of strengthening plasters.

Examinations of the urine furnished the following facts: Quantity in twenty-four hours, from twenty-seven to forty-one ounces. When the quantity was twenty-seven ounces, the specific gravity was 1008. It was one eighth albuminous. It was smoky, and frothed upon agitation. Epithelium scales desquamative, waxy, and fibrinous casts were found in abundance.

Throughout the latter half of her sickness, the patient frequently expressed herself as free from all suffering, perfectly comfortable.

Diagnosis and Treatment.—For five years past, the patient had labored under uterine troubles. These difficulties had been aggravated by the occurrence of two abortions in the last two years. Injury had just produced a third. The case was accordingly judged to be one of irritative fever with a typhoid tendency; and upon this theory the earlier treatment was based. It was not till the full examination of the urine pointed distinctly to Bright's disease, that active antagonism of the symptoms was given over.

The treatment is of little interest except so far as it teaches what was beneficial or useless in relieving symptoms. It may furnish some addition to, or corroboration of, what is known of the *juvantia* and *lædentia* in similar cases.

Milk punch and the essence of beef were the chief stimulants used. At one time, the extreme prostration demanded the exhibition of eight ounces of brandy in twenty-four hours. Various narcotics were employed to procure sleep, to relieve restlessness, or to control dyspnœa. Opium was given in several forms, and always with the effect of increasing watchfulness, or producing delirium, with this exception. In the last stages—to induce sleep, and relieve pleuretic pain, one-fourth of a grain of morphine was applied to the denuded skin. In half an hour sound sleep was produced, with a fall in the number of respirations from forty-two to twenty-five. Hyoscyamus, castor, and valerian had no effect. Asafœtida did not disappoint entirely; but the cannabis sativa answered an excellent purpose. It quieted restlessness, produced sleep, and sometimes prevented the occurrence of dyspnœa, or rendered an attack milder.

Antiperiodics, especially quinine, would sometimes prevent the paroxysms of fever, for one, or two days.

Strychnine was once given, in a very small quantity, to improve the tone of the stomach and bladder. In the twelve hours succeeding this dose there was incessant laughter and singing, in the sleep.

To relieve the head and lungs, in the latter stages, benzoic acid, acetate of potash, and tannin were tried without appreciable effect. On several occasions the carbonate of ammonia was given without any change in the symptoms.

To try the theory Frierich, as to the conversion of uruea into ammonia, in the blood, the breath was twice tested during a paroxysm of dyspnœa. No ammonia was detected.

A *post mortem* examination was made, but under the most disadvantageous restrictions as to time. Dr. WANZER and myself were unable to do more than examine the kidneys. They were of the ordinary size and shape, but softer than natural. Their color was a light purple, and their surface, as seen through the capsule very granular. Their weight was four and one-half ounces, and their specific gravity .988. It is much to be regretted that decomposition set in before specimens of these organs could be submitted to the microscope. Fifty hours after death, their structure was disorganized.

